# Control of frost formation in refrigeration applications utilizing the electrohydrodynamic technique—fundamentals, past work and prospects

**DOI:** 10.1098/rsta.2024.0364

**Published:** 2025-07-17

**Authors:** Franciene Pacheco de Sa Sarmiento, Andres Paul Sarmiento, Michael Ohadi

**Affiliations:** ^1^ Department of Mechanical Engineering, University of Maryland College Park, College Park, MD, USA

**Keywords:** frost, ice, frost control, HVAC, refrigeration, frost prevention, electrohydrodynamics (EHD)

## Abstract

Frost is an undesirable problem in energy conversion and engineering applications because it negatively affects the operating system performance by reducing the heat transfer for energy conversion systems and the coefficient of performance (COP) for refrigeration and air conditioning (HVAC) equipment. Among the various frost prevention or removal techniques, electrohydrodynamics (EHD) is an active frost prevention and removal technique that has been studied since the 1970s. This review paper clarifies the fundamentals of EHD, while offering a comprehensive review of the works published in the literature regarding both the influence of EHD on frost growth control and its effectiveness on frost removal. It is observed that while individual research works have drawn conclusions on the specifics of EHD for frost control and removal, there is no consensus in the literature on the specific effects of some of the critical parameters associated with EHD phenomena, such as the influence of electric field intensity and the use of AC and DC voltage, which can both affect frost growth. In addition, no baseline for comparison has been established, making it difficult to compare the results of various investigators. Finally, prospects and conclusions are discussed.

This article is part of the theme issue ‘Heat and mass transfer in frost and ice’.

## 1. Introduction

Frost formation is a significant problem in heating, ventilation, air conditioning (HVAC), heat pumps (HP) and refrigeration systems. Several environmental and operational variables, such as outdoor temperature, relative humidity, air velocity, air impurities and heat exchanger characteristics, can affect the frost growth rate and frost topology. If frost formation is not controlled or removed, it can affect the HVAC system operation by fully or partially blocking the inlet heat exchanger area, decreasing performance, i.e. heat-transfer rate and system coefficient of performance (COP); increasing pressure drop and pumping power; and in some severe cases causing mechanical problems to the entire system [1–3]. As the demand for energy-efficient and sustainable cooling and heating processes increases, effective defrosting methods have become crucial to enhance system reliability, reduce energy reduction consumption and optimize defrosting control [4]. Given that approximately 35% of a building’s energy consumption is used for space heating or cooling [5], it is imperative to improve the energy efficiency of buildings, specifically in HVAC and HP systems, as well as enhance the overall system efficiency, to reduce their carbon footprint and energy consumption [6].

Several strategies have been proposed to remove/avoid frost in HVAC, HP and refrigeration systems. As recently published by Sarmiento *et al.* [1], these strategies can be divided into two groups: (i) prevention or retardation of frost formation and (ii) frost buildup removal. Both frost prevention or retardation and frost removal can be subdivided into active and passive techniques. The passive techniques include surface treatment and modification, HX fin modification, and integration of PCM into the HP or HVAC system. On the other hand, active techniques include reverse cycle defrosting, oscillation and ultrasonic vibration, hot gas bypass, electric heating and electrohydrodynamics (EHD), among others [1].

Regarding active techniques, this paper is focused on EHD technology. This technique is widely used for electronic cooling, cryogenic and process industry applications, thermal and energy systems, aircraft, wind-turbine surfaces and others [7–10]. Studies regarding the effect of an electric field on frost growth began in the 1950s [11]. Frost and its interaction with an electric field were first presented by Chuang & Velkoff [12]. Apart from the traditional factors affecting ice growth over a surface, the presence of EHD leads to a non-ubiquitous interaction between frost formation and the electric field, making the system more complex and causing more variables to appear. These variables are related to the applied electric field and fluid medium and can be divided into electrode and electric field characteristics. The electrode characteristics include the gap, geometry and insulation. By contrast, the electric field characteristics are frequency, applied voltage, intensity, type of electric field (continuous or intermittent) and duration of the applied electric fields [7,13]. This review aims to analyse advances in active defrosting methods utilizing the EHD technique, clarifying their effectiveness, limitations and potential for integration into emerging heating and cooling systems. In addition, it aims to clarify the effect of select key parameters that control the EHD effect on frost growth. A detailed compilation of the studies on the physics of EHD and its effect on frost control has never been done before in the literature, making this work an original and timely contribution. For this purpose, articles related to frost growth, the influence of EHD, and EHD applications in HVAC, refrigeration and HVAC systems from the 1950s to the present were analysed. It was observed that between 2012 and 2024, there was a gap in research using EHD for the purposes studied in this article. However, the EHD technique continued to be studied and used in other areas, such as heat and mass transfer, airflow control, air filtration, solar energy and others [8,9]. This review paper is organized as follows. In §2, we present an overview of the application of plasma using the EHD technique for frost control and present the governing equations of EHD techniques. In §§3 and 4 we explore the effect of the EHD on the frost growth rate and topology, and the application of EHD to frost removal, respectively. The current status, challenges and potential directions for future research on the technology are presented in §5. Finally, in §6, we highlight the conclusions of the literature reviewed here.

## 2. Fundamentals of EHD

The concept and classification of plasma, a technique involving the application of an applied electric field, is briefly described in §2a. The theoretical basis of it and related physics are presented in §2b.

### (a) Plasma and types of discharge

Plasma is an ionized gas with a mixture of ions, electrons, radicals and neutral particles (atoms, molecules) [14,15]. According to Chen [16], plasma is a quasi-neutral gas—the total density of electrons equals the total density of ions—with charged and neutral particles that act collectively. The ionization process occurs when a gas is exposed to a high-energy source, like radiation, electrical currents or excessive thermal energy [17]. The ions and neutral particles are heavier than the electrons [14,18,19]. This means that when an electric field is applied, electrons are accelerated to higher speeds than ions in the time between collisions [16]. Depending on the energy density level, temperature and electron density in the plasma, it can be classified as thermal plasma, also called hot or equilibrium plasma, or non-thermal plasma, also called cold or non-equilibrium plasma [14,15,17]. In thermal plasma, the temperature of electrons, ions and neutral particles are similar, with an average temperature between 10 000 and 30 000 K [15]. The chemical reactions in the thermal plasma are electron-induced thermo-chemical reactions, while in the non-thermal plasma, they are mainly induced by energetic electrons [14]. In the non-thermal plasma, the temperatures of electrons, ions and neutral particles vary because the electron’s temperature is higher than that of the ions and atoms, and the plasma temperature is near room temperature [14,15]. Depending on the gas type and how the plasma is generated, the electron temperature can vary between 10 000 and 1 00 000 K [20]. Due to the action of the electric field, electrons are accelerated and energized. Through electron-impact dissociation, excitation and ionization of gas molecules, the energetic electrons transfer their energy to the gas molecules upon inelastic collision, and excited species, free radicals, ions, as well as additional electrons are produced [14]. Some examples of thermal plasma are electric arcs, ultra-high-temperature plasma-nuclear reactions and plasma jets [17]. Non-thermal plasma can be classified into dielectric barrier discharge (DBD), gliding arc, microwave discharge, corona discharge (CD) and others [17,20]. Plasma can occur naturally or can be created in the laboratory.

**Figure 1.**
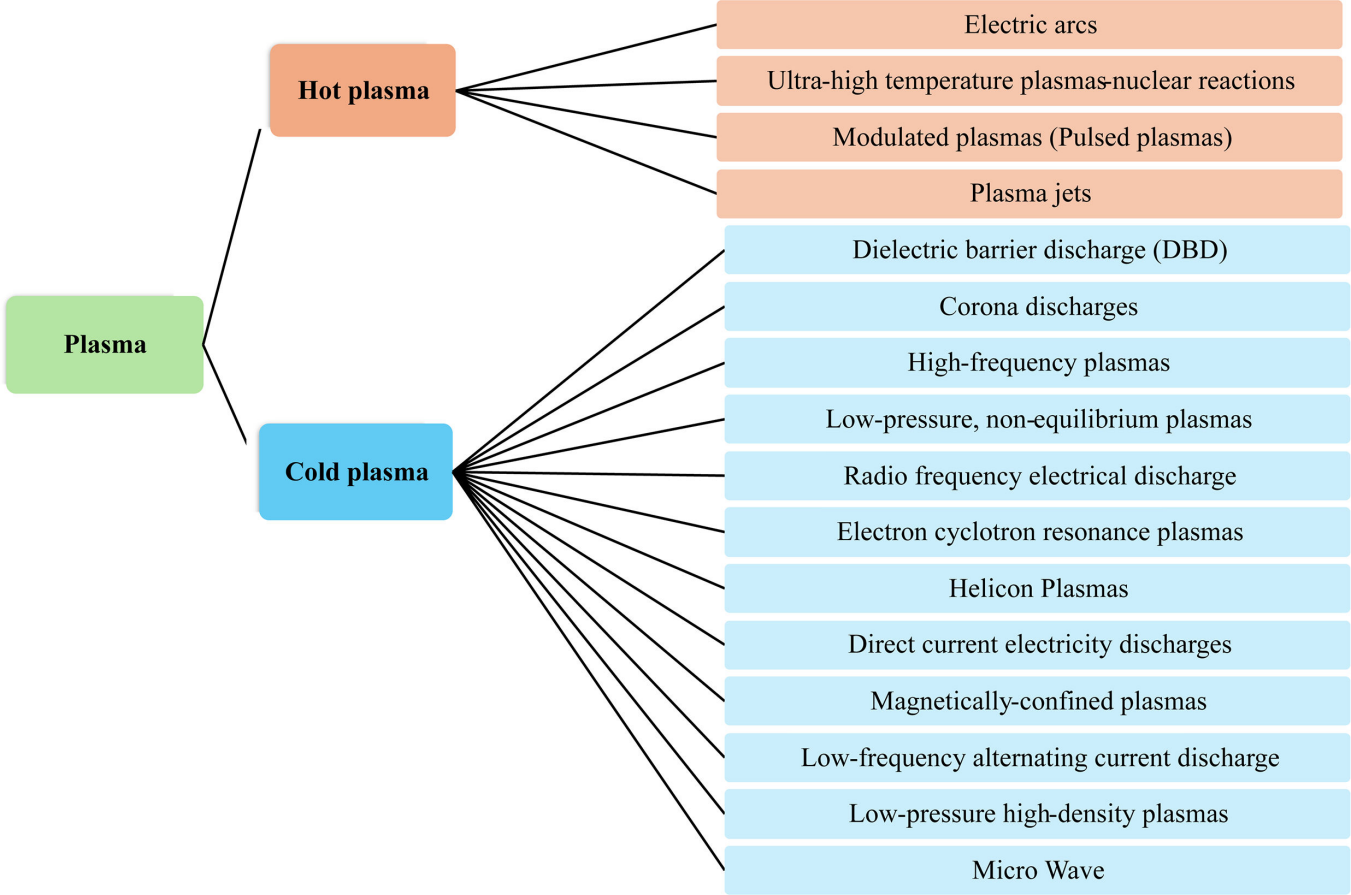
Plasma classification.

Figure 1 shows a classification of plasmas according to temperature range and type. Although several plasma types can be enumerated, this paper discusses only the DBD and the CD, as both technologies are used in the application of EHD for frost control.

**Figure 2.**
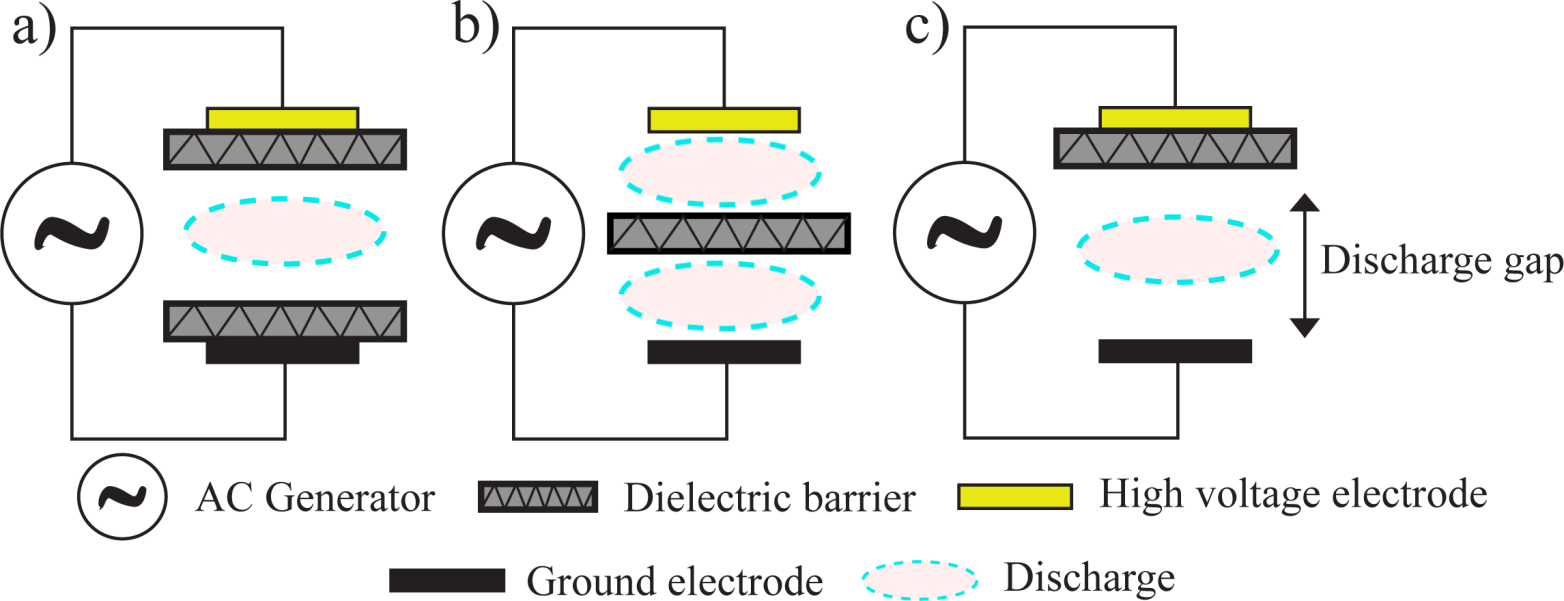
Basic DBD configurations.

The DBD is also called silent discharge or atmospheric pressure-glow [14,15,18,21]. It was first reported in 1857 by Siemens [22]. The typical configuration of DBD is two electrodes placed parallel with a gap between them, as shown in figure 2. The gap between the electrodes is on the order of a few millimetres, and a dielectric material covers at least one of the electrodes. The dielectric material could be glass, quartz, ceramic, polymer or any material with a small dielectric loss, and it is applied to prevent the formation of arcs and sparks. The applied voltage is approximately a few kV, and the DBD can be operated with AC, using frequencies between kHz and MHz, and DC current, using pulsed mode [14,15,18,20–22].

**Figure 3.**
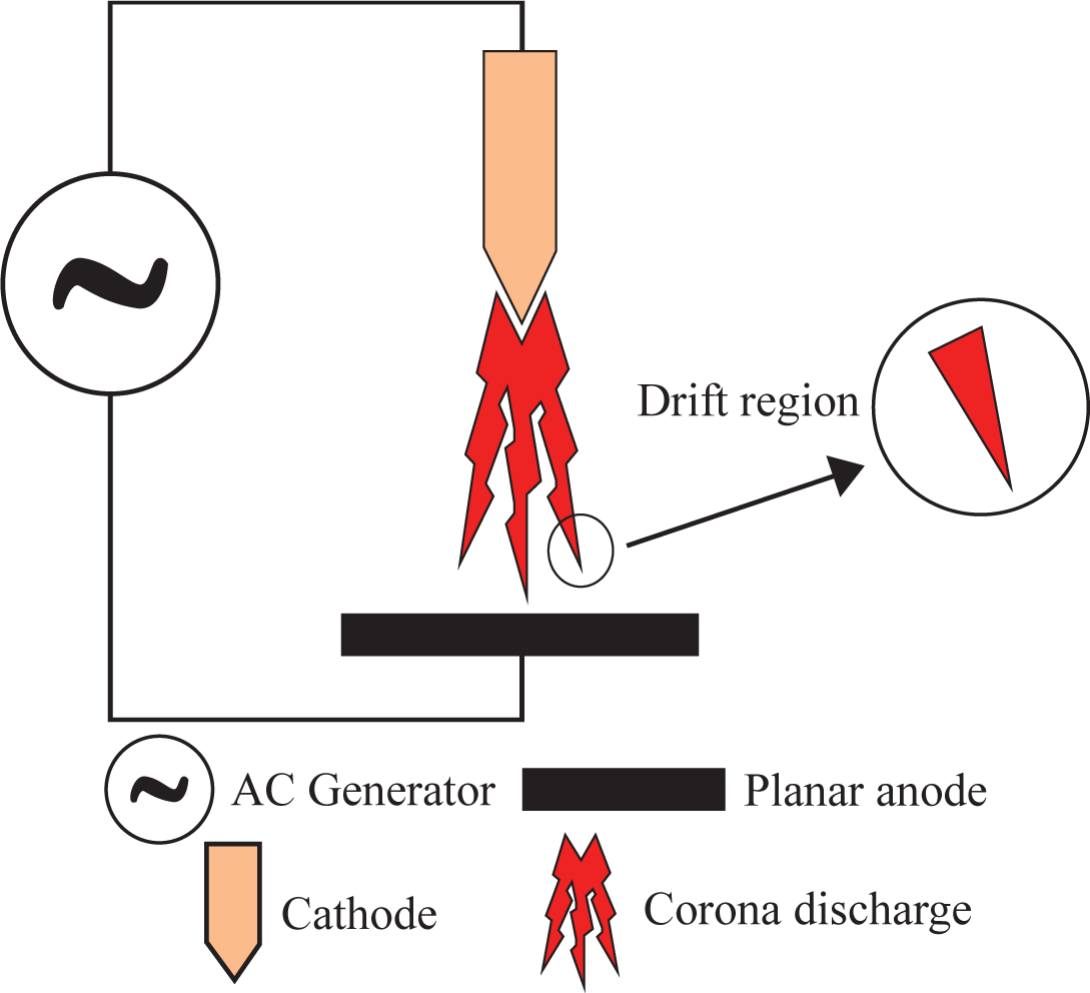
Schematic of CD.

The CD is a heterogeneous electrical discharge in a strong electric field at atmospheric pressure. Because of the strong magnetic field, the gas molecules around the electrode are ionized, generating an abundance of electrons. This abundance generates an additional electric field, creating an ionization zone that produces a discharge around the electrode [17,20]. Two electrodes are commonly applied in the CD (figure 3): one with a high curvature and the other with a low curvature, such as a plate or cylinder. The type of CD depends on the electrode configuration (geometry and polarity of the active electrode), the type of gas and the voltage used [17,23]. The voltage used can be AC or DC [23–25]. Depending on the polarity in the active electrode, the CD can be positive or negative. More information about positive and negative CDs can be found in [23,26].

The typical characteristic parameters of DBDs and CDsd can be found listed in table 1, highlighting the electric field values, degree of ionization and electron density, among others.

**Table 1.** Typical characteristic parameters of the DBD and CD [20].

parameter	DBD value	corona value
electric field (V cm^−1^)	0.1−100	0.5−10
degree of ionization	~10^–4^	small, variable
reduced electric field (Td)	1−500	2−200
electron density (cm^−3^)	10^12^−10^14^	10^9^−10^13^
average electron energy (eV)	1−10	3.5−6

### (b) The EHD body force

The energy in an electric field can be expressed by using Maxwell equations as follows:


(2.1) $$U=\frac{\varepsilon_{0}}{2}\int E^{2}dv,$$

where U is the field energy, $\varepsilon_{0}$ is the dielectric permittivity of vacuum and v is the volume. The electric displacement vector can be defined as


(2.2) $$\vec{D}=\varepsilon_{0}\;\vec{E}+\vec{P},$$

where $\vec{E}$ is the electric field and $\vec{P}$ is the polarization field defined as the dipole moment per unit volume. Polarization force arises from the displacement of charges resulting an applied external field. In most cases, the polarization is proportional to the applied electric field and can be written as


(2.3) $$\vec{P}=\varepsilon_{o}\chi \vec{E},$$

where χ is the electric susceptibility. Substituting equation (2.3) into equation (2.2), one obtains


(2.4) $$\vec{D}=\varepsilon_{o}\;\vec{E}+\varepsilon_{o}\;\chi \vec{E}=\varepsilon_{o}\left(1+\chi \right)\vec{E}.$$

By setting k=1+χk equal to 1+χ, equation (2.4) is reduced to


(2.5) $$\vec{D}=k\varepsilon_{o}\vec{E},$$

where k is known as the dielectric constant and measures the extent of the polarization produced within a material by an applied electric external field. Assuming that the dielectric medium is maintained stationary, no work is done on mechanical constraints, and the field energy, equation (2.1), can be written as


(2.6) $$U=\frac{1}{2}\int \vec{E}\cdot \vec{D}\;dv.$$

Considering a dielectric fluid that undergoes a small displacement, δ on the *n*-axis given by a volume force ${\vec{f}}_{e}$, created by an electric field, the resulting free energy is given by


(2.7) $$\delta U=-\int {\vec{f}}_{e}\cdot \delta n\;dv.$$

As shown by Stratton [27], the electric body force per unit volume acting on a dielectric medium placed in an electric field can be written as


(2.8) $${\vec{f}}_{e}=\rho_{c}\vec{E}-\frac{1}{2}E^{2}\nabla \varepsilon +\frac{1}{2}\nabla \left[\rho E^{2}{\left(\frac{\partial \varepsilon }{\partial \rho }\right)}_{T}\right],$$

where ε is the dielectric permittivity of the fluid, ρ is the mass density, $\rho_{c}$ is the electric field space charge density, *T* is the temperature and E is the applied electric field strength. The electric field space charge density is the number of free charges in the fluid per unit volume.

The first term on the right-hand side of equation (2.8) represents the electrostatic force (Coulomb) and the electrophoresis force; the second term refers to the dielectrophoretic force, and the last term is the electrostrictive force. Electrostatic and electrophoresis forces result from the free charges within the dielectric fluid, where these forces are responsible for the alignment and rotation of fluid molecules in the direction of the electric field. The dielectrophoretic force is related to the translational movement of neutral particles due to the polarity of the electric field, resulting from the interaction between the particle’s dipole and the spatial gradient of the electric field and/or dielectric permittivity. Finally, the electrostrictive force represents a volume force whenever an inhomogeneous electric field exists within the dielectric and a variation of the dielectric permittivity with density.

Dielectrophoretic force can be expressed as


(2.9) $$\frac{1}{2}E^{2}\nabla \varepsilon =\frac{1}{2}E^{2}\left[{\left(\frac{\partial \varepsilon }{\partial \rho }\right)}_{T}\nabla \rho +{\left(\frac{\partial \varepsilon }{\partial T}\right)}_{\rho }\nabla T\right].$$

The direction of the force depends on the direction of the increase of ε. If the fluid is non-polar, the second term on the right-hand side in equation (2.9) will be zero, and therefore, equation (2.9) can be simplified by the Clausius–Mossotti law. This simplification cannot be applied if the fluid is polar, where the electrical permittivity is a function of temperature and density.

**Figure 4.**
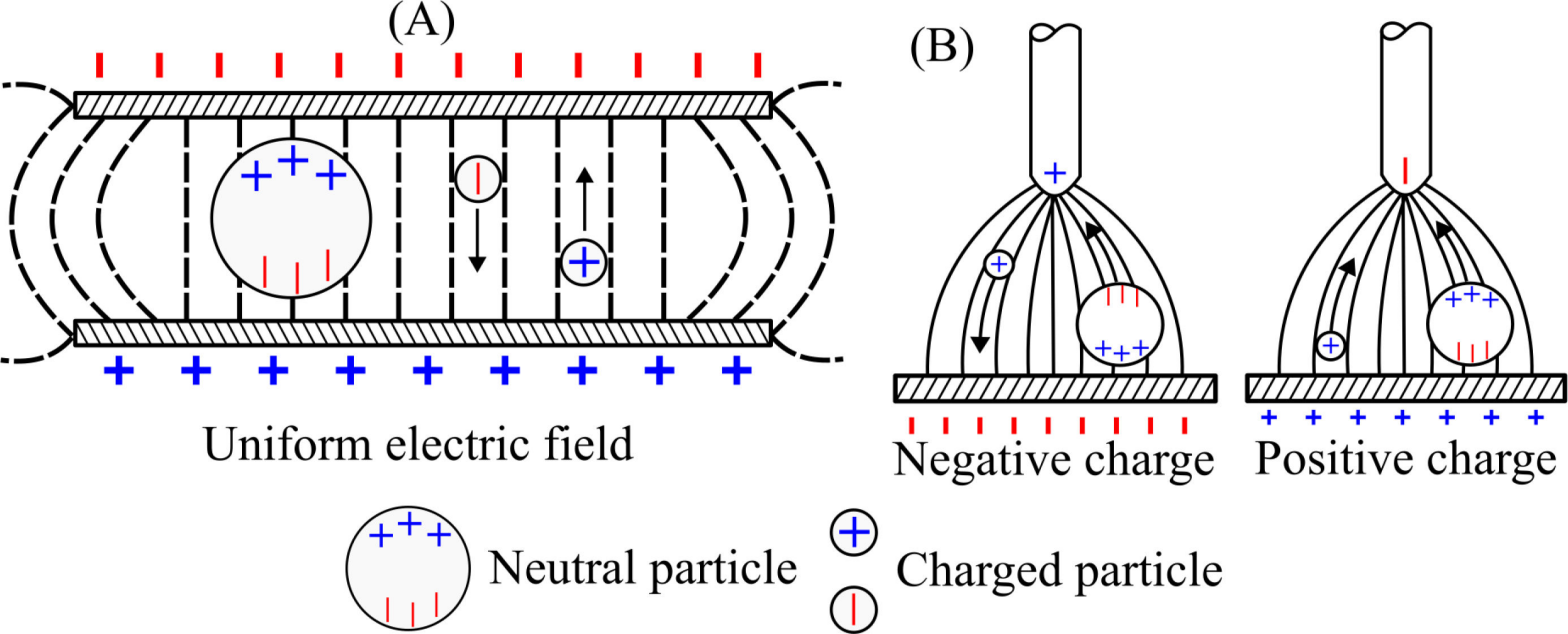
(A) The behaviour of charged and neutral particles in a uniform electric field; (B) the behaviour of charged and neutral particles in non-uniform electric fields.

The electric field can be uniform or non-uniform. A charged particle in a uniform electric field migrates to an electrode of opposite polarity. By contrast, a neutral particle is polarized but does not produce translational motion, as shown in figure 4*A*. On the other hand, in a non-uniform electric field, the neutral particle presents a translational motion due to the difference between the positive and negative charges at the two ends of the dipole. This difference creates a force on the neutral particle that moves this particle towards the stronger field, as shown in figure 4*B*. The charged particle has the same behaviour as in the uniform field.

For further information on the EHD formulation, please refer to [9,19,28].

## 3. Frost formation under EHD presence

**Figure 5.**
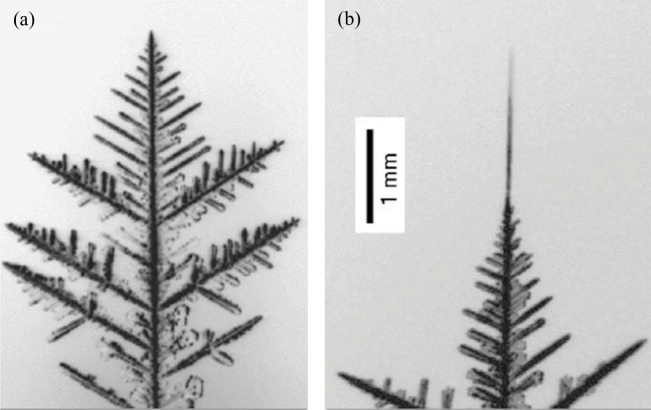
Ice-crystal dendrites grown from supersaturated air at −15°C without the electric field presence (a), and with the presence of electric field (b). From Libbrecht & Tanusheva [29], used with permission from the American Physical Society.

It is known in the literature that the behaviour of ice growth under EHD conditions is different when EHD is not applied. Side branches normally characterize ice growth without EHD, see figure 5*a* [30]. On the other hand, the ice growth under the effect of EHD is needled-shaped, with longer and thinner crystals, as shown in figure 5*b* [29–31] . This is because the electric field changes the saturation pressure and the driving force of the phase change [31]. More information about ice growth without EHD can be found in [1].

In 1953, Shaefer [11] was the first author to report the ice growth under an electric field. Shaefer [11] used a DC electric field on supercooled clouds and observed observed rapid ice growth in the form of whisker-like conglomerates at high electric fields, with the crystals growing in the direction of the electric field’s force. Magono & Sekiya [32] reached the same conclusion as Shaefer, that ice crystals grow faster when subjected to an electric field. They also found that the ice growth follows the line of force in the electric field.

**Figure 6.**
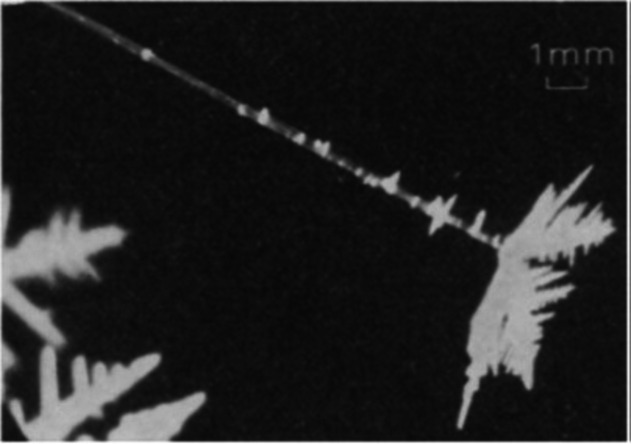
An ‘electric’ needle growing at −4°C under the influence of an electric field. From Bartlett *et al*. [30] used with permission from Springer Nature.

Marshall & Gunn [33] observed chaotic frost crystal growth with many irregular branches of small cross-sections under the influence of relatively weak electric fields. In 1963, Bartlett *et al.* [16] reported for the first time a growth of needle ice, called ‘electric’ needles, when an electric field was applied in excess of 500 V cm^−1^ (figure 6); the authors found similar results for both polarities. Bartlett *et al.* [16] also observed that the electric crystals fractured spontaneously and travelled towards the other electrode.

Maybank & Barthakur [34] observed that no change occurred in the growth rate and shape of the ice crystal for fields below 200 V cm^−1^. However, when the field was above this value, an increase in the growth rate was observed, reaching 150 µ s^−1^ when the electric field was 1500 V cm^−1^. The number of crystals also increased, and they were thinner and more fragile. The authors observed that the ambient temperature inside the chamber affected the appearance of the ice-crystal growth. At temperatures above −12°C, opaque sprouts grew rapidly as thin needles, while at temperatures below −12°C, the long, thin needles were replaced by even finer filaments that fractured readily.

**Figure 7.**
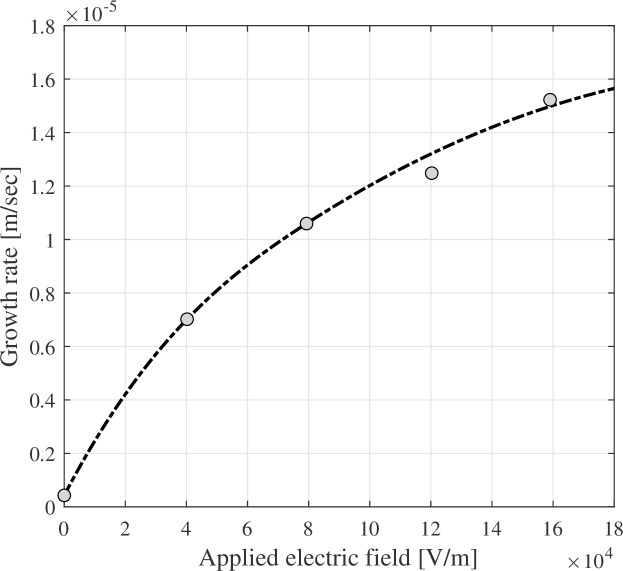
The ice-crystal growth rate as a function of an applied electric field. Data from Crowther [35], used with permission from the Elsevier.

Crowther [35] observed experimentally the growth of ice crystals from the vapour in a continuous diffusion cloud chamber under an electric field presence. Crowther concluded similarly to Bartlett *et al.* [16], finding that ice-crystal growth was independent of field polarity and that the ice crystal travelled to the nearest electrode after the fracture. Crowther also showed a growth rate curve based on the applied electric field strength (figure 7). The growth rate increased with the increased electric field, and needle-like crystals formed.

In 1972, Motoc [36] studied dendritic growth with and without the influence of the electric field. In this study, AC and DC electric fields were utilized. Under a DC electric field, the ice crystal grew in the field direction, and crystalline needles were observed. In the presence of the AC electric field, fields below 700 V cm^−1^ did not influence the ice-crystal growth. Above this value, the dendritic growth was directly parallel to the field, thickening the central stems.

Ma & Peterson [31] developed equations based on fundamental thermodynamic principles and phase-change theory to express the saturation pressure and the critical radius for ice-crystal nucleation under the influence of electric fields. They assumed that the vapour exhibits ideal-gas behaviour at constant temperature. According to the authors, increasing the electric field decreased both saturation pressure and critical radius and resulted in the formation of needle frost.

Libbrecht & Tanusheva [29] extended the solvability theory for free dendrite growth under an electric field and experimentally tested the theory to observe the growth. They observed that the dendrite growth is faster with the presence of an electric field than without. When the electric field was applied, the tip velocity increased from 3 µm s^−1^ to 70 µm s^−1^. This increase in tip velocity resulted in the rapid growth of branchless ice needles, as shown in figure 5. In 2002, Libbrecht *et al.* [37] used new experimental data and a new model to describe the diffusion-limited growth of dendritic needle crystals from the vapour phase. They found the existence of a threshold potential above which the needle growth presented a morphological instability. They also found that the tip velocity increased approximately linearly with the vapour supersaturation and with the applied potential.

**Figure 8.**
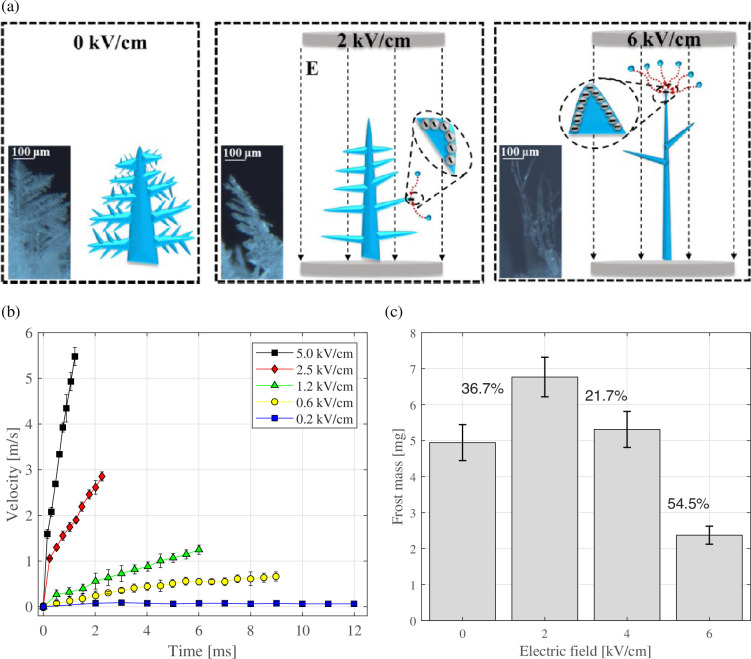
(a) Schematic diagram of frost crystal morphology under different electric fields, (b) the jumping speeds of frost crystals at different electric field strengths, (c) and frost mass on the frozen droplet at different electric fields. Data and figures from Xu *et al.* [38], used with permission from Elsevier.

Recently, Xu *et al.* [38] studied experimentally, through visualizations, the evolution of frost on a frozen droplet under an electric field. They found that the characteristics of frost growth and jumping were affected by the electric field. Figure 8*a* shows the influence of the electric field on the frost crystal morphology. With the increase in the electric field, the frost crystals become thinner and taller, with fewer lateral branches, compared with the case where the electric field is absent (0 kV cm^−1^). The jumping speed was also influenced by the electric field (figure 8*b*): as the electric field increased, the jumping speed also increased. They observed that with the increase in the electric field, there was first an increase in frost mass (*E* = 2 kV cm^−1^) and then a reduction that reached 54.5% when the applied field was 6 kV cm^−1^ (figure 8*c*).

Based on the above studies, most of the previous works conclude that the application of an electric field causes the ice growth rate to increase with the increase in the electric field, thus modifying the frost/ice-crystal structure. This increase in the growth rate causes the ice crystal to become thinner and more fragile, which makes it easier to fracture/remove. Furthermore, it was observed that the direction of ice-crystal growth depends on the field polarity; with DC fields, the crystal grows towards the field, while with AC fields, the crystal grows parallel to the field. Another factor that influenced the crystal growth was the intensity of the applied electric field. For voltages below 200 V cm^−1^, with DC, no influence on the field was observed, while for AC, the corresponding threshold value is 700 V cm^−1^.

## 4. EHD effect on frost control/removal

The EHD literature can be divided into two groups (i): studies in which the electrode was placed inside the plate, meaning that the electrode that generates the electric field is placed inside the plate [39–43], and (ii) studies in which the electrode was placed outside of the plate, e.g. the electrode is placed on another plate or next to the surface to be defrosted [38,44,45].

In 1971, Chuang & Velkoff [12] examined frost mass on a flat plate under natural convection. The frost mass increased when the corona current was increased, and the frost mass deposition rate decreased when the current decreased. They observed an increase of approximately 200% in frost mass when the corona current was equal to 200 *µ*A.

**Figure 9.**
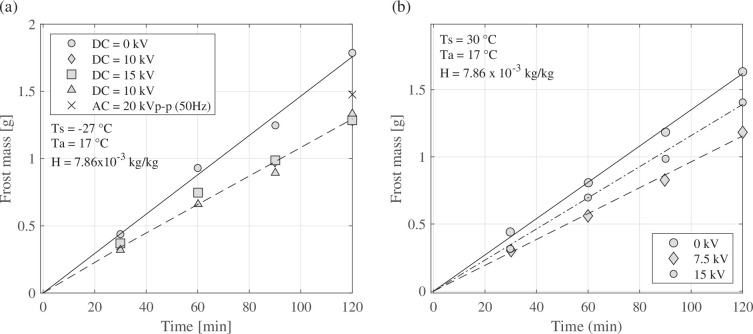
The influence of electric field intensity on frosting weighs for (a) a copper plate under AC and DC electric fields and (b) for a PTFE surface under DC electric fields. Data from [46].

Munakata *et al.* [46] investigated the electric field effect on frost patterns under natural convection on a flat plate using a mesh electrode. They applied an AC and DC electric field on a copper surface and a DC electric field on a polytetrafluoroethylene (PTFE) surface. The authors compared all the results with the base case (without the electric field). For the copper surface under DC, the reduction in frosting weights was approximately 25%, while for the surface under AC, the reduction was approximately 16% (figure 9*a*). However, the polarity effect was not observed. For the PTFE surface, the frosting weights decreased by approximately 30% (using 7.5 kV) compared with the case with no electric field (figure 9*b*). Munakata *et al.* [46] concluded that surface wettability weakly affected frosting formation and did not affect electrical frosting prevention.

In 1995, Blanford *et al.* [47] used a CD to study the effect of an electric field under forced convection in a finned heat exchanger. They reported that when the CD current was up to 20 *µ*A, the frost mass reduction decreased by 20%. However, when the CD current was equal to 120 *µ*A, the frost mass increased by 220%.

Mishra *et al.* [48] experimentally investigated the effect of electric fields on a flat plate under natural convection. They varied the humidity, air temperature, voltage and plate temperature. The authors found a reduction in the frost mass of up to 30% when the applied electric voltage was 18 kV.

Bloshteyn *et al.* [49], in 1999, used an insulated and bare-wire electrode on a vertical cold plate to control frost growth. They applied a DC electric field. The authors reported that the use of the insulated electrode avoided the current leakage and sparking, and consequently, the power consumption was reduced. Using an insulated electrode, the frost reduction was approximately 30%. On/off cycling was used to reduce the accumulated charge in the insulated electrode and to increase the frost reduction.

Molki *et al.* [50] studied the effect of an intermittent electric field on a vertical surface under natural convection. The authors found that the intermittent electric field quickly destroyed the developed frost crystals. However, initially, the intermittent electric field removed less frost than the continuous field. This situation was reversed after approximately 1 h. If the frequency of the field is increased, the effectiveness of frost removal is also increased.

In 2002, Ohadi & Anad [51] experimentally investigated frost formation under the influence of AC and DC electric fields under natural and forced convection. They tested a vertical and horizontal flat plate and a continuous and intermittent electric field. The authors observed that for the vertical plate under a continuous DC electric field, the frost mass decreased up to 30.8%, while under an intermittent DC electric field, the reduction was 34.7%. For the horizontal plate under a DC electric field, the frost mass reduction was between 4.5% and 28%, depending on the electrode applied and the polarity. When a continuous (applied during 2 h) AC electric field was applied to the vertical plate under natural convection, the frost mass accumulated on the surface increased between 14.5% and 44%. However, when an intermittent electric field was applied (1 min after 30, 60 or 120 min), the frost mass on the surface decreased up to 13.5%.

**Figure 10.**
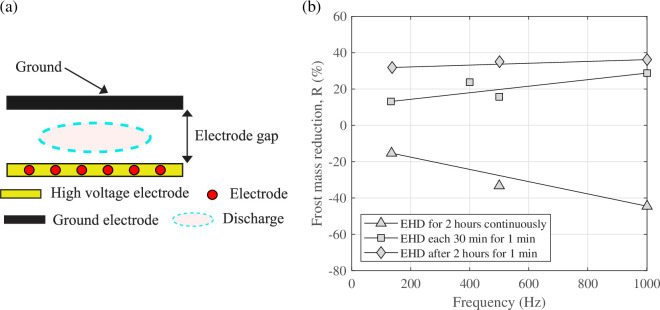
(a) Schematic of electrode gap (b) and influence of the applied AC voltage frequency on frost mass reduction (R) Ta = 0°C, Tp = −30°C, *L* = 5.0 mm, *V* = 14.5 kV. Data from Tudor *et al.* [39], used with permission from Taylor and Francis.

Tudor *et al.* [39] investigated the effects of AC and DC electric fields and forced convection on frost formation on a horizontal downward-facing flat plate. The electrodes were placed inside the flat plate, and the electrode gap was 8 mm for AC electric field and 5 mm for DC electric field (see figure 10*a*). The conditions tested for the AC electric field were the cold plate temperature equal to 0°C and the air temperature equal to −30°C. For the DC electric field, the conditions tested were the air temperature equal to −10°C, the plate temperature equal to −30°C and −20°C, and the relative humidity equal to 85% and 90%, respectively. The reduction in frost mass was up to 10% for the DC electric field when the voltage was equal to 7.5 kV and up to 36% for the AC electric field when the voltage was equal to 14.5 kV (figure 10*b*). The results of forced convection were similar to those of the AC electric field.

**Figure 11.**
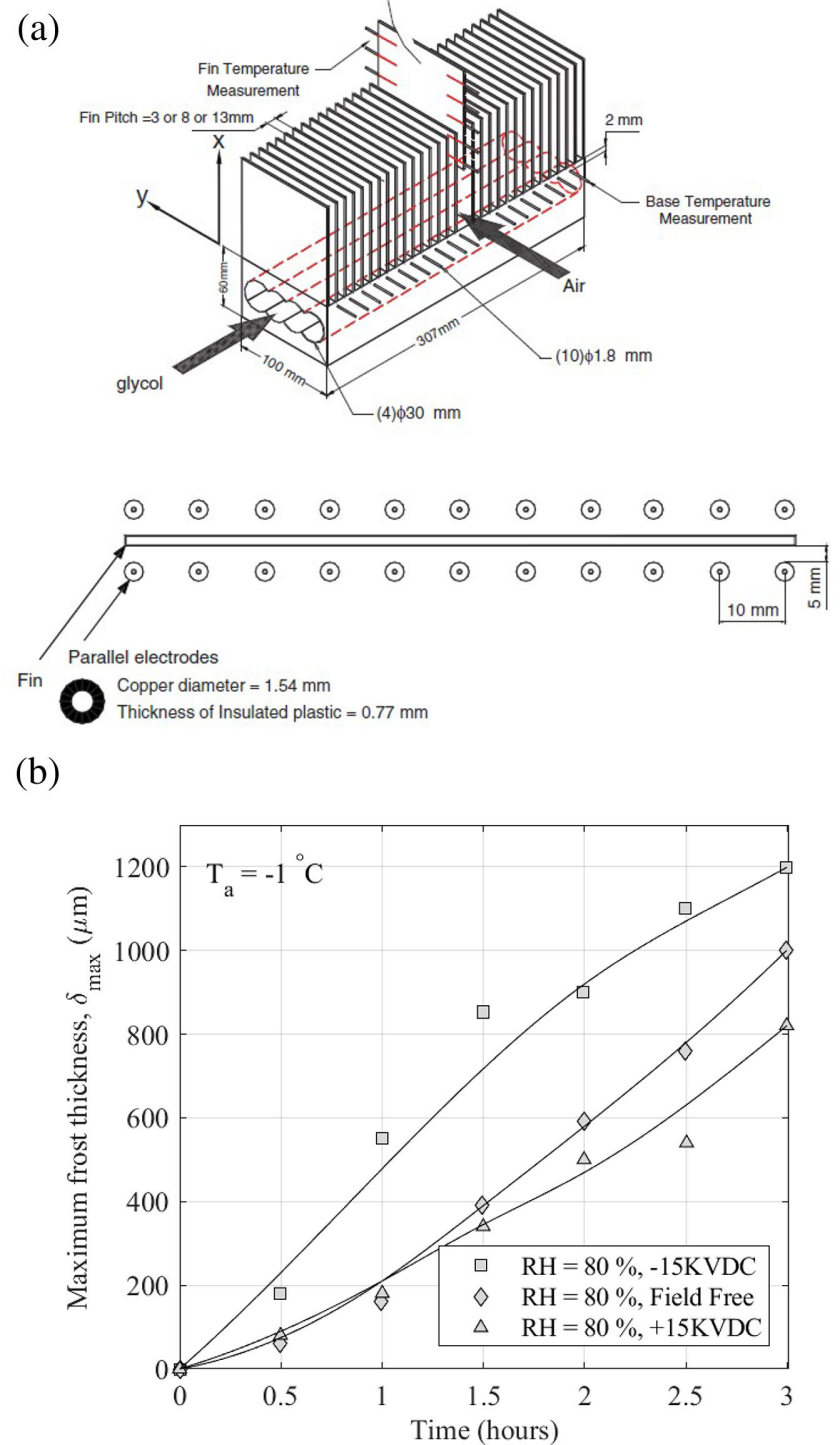
(a) Schematic of the test section and (b) the maximum frost thickness as a function of time under the influence of electric polarity. Data from [41]. From Wang *et al.* [41], used with permission from Elsevier.

Wang *et al.* [41] investigated the influence of polarity on DC electric fields in natural convection on an array of vertical surfaces which were aligned perpendicularly to the airflow direction (figure 11*a*). They observed that the presence of EHD caused the frost structures to be skinny and fragile in shape. They found that the polarity of the field affected the frost structure. The negative polarity showed a thinner structure and higher break-off frequency of the ice column than the positive polarity. The electric field also influenced the frost thickness, as shown in figure 11*b*. The negative polarity produced a frost thickness 30–50% greater than the positive polarity.

**Figure 12.**
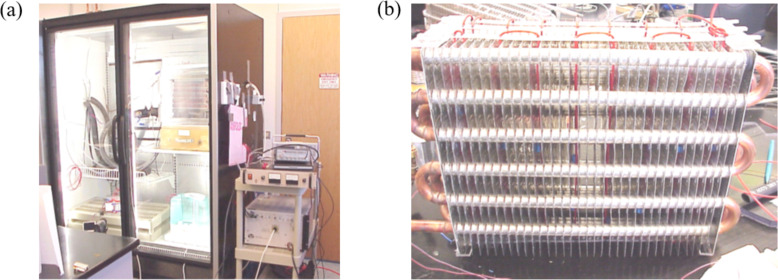
Freezer test chamber (a) and test evaporator with wire electrodes (b). Reproduced with permission from the authors [52].

Tudor *et al.* [44] applied a DBD technique to control the frost on evaporator coils. They tested two evaporators: a small-scale laboratory test module and a full-scale supermarket evaporator (figure 12*a*). The electrode was placed in the gap between two fins where frost accumulated (figure 12*b*). The authors applied an AC voltage of 7−12 kV with frequencies ranging from 700 to 3000 Hz. The results showed that the time of defrost and the energy consumption using DBD were less than that using electrical heating.

**Figure 13.**
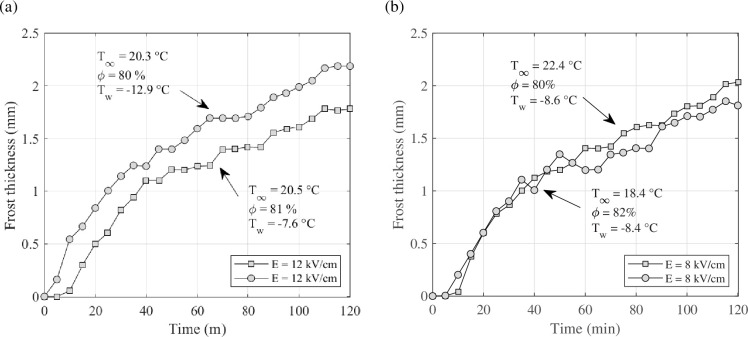
Results for the influence of cold plate temperature (a) and the ambient temperature (b) on frost-layer thickness. Data from [42].

Zhang *et al.* [42] investigated the influence of the DC electric field, plate temperature and ambient temperature on the mass and thickness of frost on a cold vertical plate. Natural convection with the electric fields was tested in these experiments. The results of applying EHD showed that the cold plate temperature affected the frost-layer thickness more than the ambient temperature (figure 13). However, the frost mass was not influenced by the cold plate temperature. On the other hand, the ambient temperature significantly affected the mass frost deposition.

Tudor & Ohadi [40] applied an AC electric field on a cold plate and compared the results with the DC electric field results presented in [13]. The AC electric fields were applied continuously and at intervals. They found that when the AC was continuously applied, the mass of frost in the plate increased, while when the electric fields were applied at time intervals, the frost mass was reduced. Depending on the frequency and the time intervals, the frost mass was reduced by up to 46%. They concluded that most of the crystals were broken in the first seconds when the electric field was applied since the crystals that were growing were subjected to the field for only 10 s or less.

**Figure 14.**
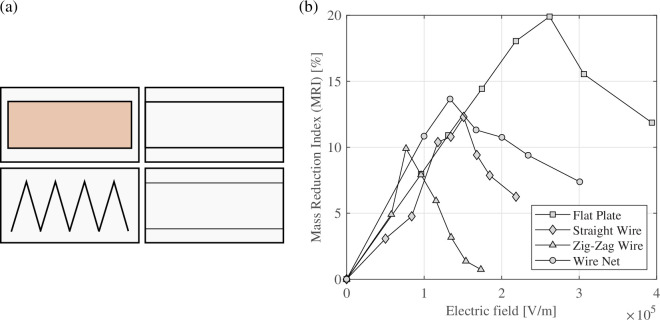
Layout of electrodes (left) and mass reduction index as a function of the electric field intensity for all the tested electrodes (*t*_A_ = 10°C, RH = 75%, *t*_S_ = −3°C, *v* = 3 m/s and *t* = 7200 s) (right). Data from Joppolo & Molinaroli [53] used with permission from ASHRAE.

In 2009, Joppolo & Molinaroli [53] used a copper flat surface with four different electrode geometries: flat surface, straight wire, zig-zag wire and a wire net (30 mm × 30 mm) (figure 14). They used an upward-facing flat plate and a DC electric field in the experiments to investigate frost formation and accumulation. They observed the influence of electric field intensity and uniformity, air velocity, cold surface temperature and test duration. Related to the influence of the electric field, the mass reduction index increased with intensity up to a maximum and then started to decrease. The same behaviour was found for all electrode geometries. Depending on the test duration, the mass reduction index could be up to 26% when a flat plate was used. The second-best mass reduction index was produced by the wire net, followed by the zig-zag wire, and finally, the straight wire presented the worst result. This sequence of results was observed for all results.

Joppolo *et al.* [43] investigated the influence of DC electric field intensity in a fin and tube evaporator. The frost mass behaviour, air side pressure drops, evaporation temperature and air velocity were also investigated. They found that the frost mass decreased with increasing intensity, but it had a maximum after which it began to grow again. This behaviour was similar to that found by Joppolo & Molinaroli [53] (figure 14*b*). Joppolo *et al.* [43] observed that the evaporation temperature and air velocity affected the frost mass reduction. They found a reduced mass frost of approximately 12.75% due to air velocity and 10.5% to evaporation temperature. The energy saving was 11.5% when compared with the same system without EHD.

Table 2 lists a summary of studies in which EHD was used for frost control/removal.

**Table 2.** Summary of the literature for EHD.

authors	geometry	electric fields	electrode	results
Chuang & Velkoff [12]	flat plate	—	corona	the frost mass was increased by 200% when the corona current was 200 µA.
Munakata *et al.* [46]	flat plate: copper and PTFE	AC/DC	mesh electrode	the frosting weights were reduced by approximately 25% and 16% when DC and AC were applied, respectively, for a copper plate. For the PTFE, the frost weight reduction was approximately 30%.
Blanford *et al.* [47]	finned heat exchanger	—	corona discharge	when the corona discharge current was 20 *µ*A, the frost mass reduction of 20%. However, when the corona discharge current increased to 120 *µ*A, the frost formation mass increased by 220%.
Mishra *et al.* [48]	flat plate	—	—	the frost mass reduction was up to 30% when the electric voltage was 18 kV.
Bloshteyn *et al.* [49]	flat plate	DC	bare-wire and insulated electrodes	the frost reduction was approximately 30%. The on/off cycling reduced the frost more efficiently than the continuous cycles.
Molki *et al.* [50]	vertical surface	—	—	the intermittent electric field removed frost more effectively than a continuous electric field.
Ohadi & Anad [51]	horizontal and vertical flat plate	AC/DC	parallel wire, zig-zag wire and net	for DC electric field and vertical orientation, the frost mass reduction was approximately 31% for continuous application and approximately 35% for intermittent electric field. When the flat plate was oriented on horizontal, the frost mass reduction was 4.5%.
Tudor *et al.* [39]	flat plate	AC/ DC	inside/12 parallel insulated wires	frost mass reduction was up to 10% and 36% when DC and AC electric fields were applied, respectively.
Wang *et al.* [41]	array of vertical surfaces	DC	inside/22 parallel insulated wires	the frost thickness increased 20% when negative polarity was applied and reduced 15% when positive polarity was applied.
Tudor *et al.* [44]	section fin geometry and evaporator	AC	DBD	the defrosting time and energy consumption were less than those resulting from electrical heating.
Zhang *et al.* [42]	cold vertical plate	DC	inside/24 parallel wires	the mass of frost was reduced 18% compared with without EHD.
Tudor & Ohadi [40]	cold plate	AC	inside/12 parallel insulated wires	the frozen mass can be reduced up to 46% depending on the frequency and time applied.
Joppolo & Molinaroli [43]	upward-facing flat plate	DC	Four different geometries: flat plate, straight wire, zig-zag wire and a wire net (30 mm × 30 mm)	the mass reduction index increased with the intensity up to a maximum and then started to decrease. The mass reduction index could be up to 26% when a flat plate was used.
Joppolo *et al.* [43]	fin and tube evaporator	DC	insulated wire	the evaporation temperature and air velocity affected the frost mass reduction.

## 5. Current status, challenges and the future direction of technology

Over the last two decades, several experimental evaluations have been developed, highlighting the potential of EHD-based frost removal/avoidance in surfaces and heat exchangers. However, none of the previous studies have demonstrated the method at a meaningful scale and under actual environmental/operational test conditions. While this is a significant shortfall, it presents a research opportunity to unlock the full potential of these active, online/on-demand techniques. Furthermore, it was observed that in the literature, there is no universal consensus on the role of some of the key parameters that influence the effectiveness of the EHD technique in frost prevention/control. Examples include the effect of AC versus DC applied electric field, intermittent versus continuous electric field and field polarity. For example, on the role of AC versus DC, some studies report that using DC current reduces frost mass formation more than AC [46] and [51]. On the other hand, Tudor *et al.* [39] claim that using AC current reduced the frost mass more than the DC current. Regarding the application of intermittent versus continuous field, Tudor & Ohadi [40] and Ohadi & Anad [51] report that the continuous AC field application increased the frost mass formation, while intermittent application showed a reduction. In a separate study, Bloshteyn *et al*. [49] and Ohadi & Anad [51] report that when a DC current is applied, the intermittent applied field presents better results than the continuous field. On the other hand, Molki *et al.* [50] showed that applying the continuous field was more efficient in the first hour of removal and that applying an intermittent field was more effective after this period. The polarity of the electric field also presents divergence in the reported works. For example, Munakata *et al.* [46] did not observe any influence of polarity in their experiments, whereas Wang *et al.* [41] showed that depending on the applied field polarity, the frost structure and frost thickness can increase or decrease. Another point of divergence in using EHD is in relation to the influence of the increase in the electric field intensity. In a study by Joppolo *et al.* [43], increasing the electric field intensity increases the reduction in frost mass formation, which is contrary to what was observed by Chuang & Velkoff [12], where with increasing the corona current, there was an increase in frost mass formation. In a separate study, Joppolo & Molinaroli [45] described that with the increase in field intensity, there was a reduction in frost up to a certain maximum point. After that, an increase in frost will occur. These findings suggest the need for additional modelling and experimental work to characterize/quantify the effects of key controlling parameters on the control of frost formation with the EHD technique. Additional geometric, flow-field and phase change-related parameters might need to be defined to more precisely define the experimental conditions for the various studies while facilitating a better understanding of the physics involved. Accordingly, the authors suggest the following research gaps present opportunities for additional research in the respective fields.

(a) The influence of variables/parameters, such as gap and electrode type, the application time of the electric field (continuous or intermittent) and the intensity of the electric field in the frost removal/formation, should be investigated.
(b) Despite much research on EHD-based techniques, no standard baseline for comparison has been established. Experimental evaluations under consistent conditions should be conducted, such as air inlet temperature and relative humidity, as well as well-defined electric field variables/parameters.
(c) Numerical models should be developed, in which the effect of the EHD electric field is coupled with mass transfer and fluid flow, to understand the interaction between these variables and frost growth rate and topology.
(d) The EHD-based frost method should be implemented on practical heat exchangers/evaporators to verify the effect of the electric field in real-world applications.
(e) Combining the EHD technique with frost tracking methods such as artificial vision or artificial intelligence can help reduce energy consumption by giving predictions about the time to start or shut down the frosting control system.

## 6. Conclusions

Frost formation in HVAC, HP and refrigeration systems is a ubiquitous problem. This is especially common when the operation of the system has lower surface temperatures and moderate to high relative humidity conditions. This paper offers a review of EHD-based frost prevention and frost removal in HVAC systems. The study began with an overview of active and passive frost control and removal techniques. In §2, we presented a brief description of the EHD technique, focusing on the physical phenomena involved. In §§3 and 4, the influence of EHD on frost growth rate and topology was presented. Finally, the current status, challenges and potential future directions for actively controlled frost prevention/removal technologies were presented in §5.

Based on this comprehensive literature review, the following conclusions can be drawn:

(1) A number of parameters in an applied the electric field influence the frost growth, including: electric field intensity, polarity, electrode gap distance, type of electrode, whether the electric field is continuous or intermittent and the time applied.
(2) The electric field influences frost growth depending on the applied field. For low-intensity electric fields, the growth of the ice crystal is less influenced, but at a certain intensity level, the morphology of the ice crystals begins to change, and the crystal becomes thinner and taller, with fewer lateral branches.
(3) The electric field also influences the tip velocity of the crystal growth, which increases as the electric field increases. This, in turn, leads to the crystal becoming thinner and more fragile.
(4) The polarity of the electric fields affects the growth direction and the frost mass reduction. For DC fields, the ice crystals grow in the direction of the field and can reduce the frost mass by up to 36%, based on the reported experimental results. For AC fields, the ice crystals grow directly parallel to the field and can reduce the frost mass by up to 46%.
(5) The intensity of the electric field can reduce or increase the frost mass, as demonstrated in various studies cited in this article.
(6) There is no consensus in the literature on which type of application of the electric field, continuous or intermittent, presents better results in removing the frost mass from the cold surface.
(7) Additional studies aimed at the EHD control of frost are needed to settle several uncertainties and inconsistencies among some of the previous studies. The prospects of the technique otherwise remain very promising.

## Data Availability

This article has no additional data.
